# VIRE: a metagenome-derived, planetary-scale virome resource with environmental context

**DOI:** 10.1093/nar/gkaf1225

**Published:** 2025-11-29

**Authors:** Suguru Nishijima, Anthony Fullam, Thomas S B Schmidt, Michael Kuhn, Peer Bork

**Affiliations:** Molecular Systems Biology Unit, European Molecular Biology Laboratory, 69117 Heidelberg, Germany; Life Science Data Research Center, The University of Tokyo, 277-0882 Chiba, Japan; Molecular Systems Biology Unit, European Molecular Biology Laboratory, 69117 Heidelberg, Germany; APC Microbiome and School of Medicine, University College Cork, Cork, Ireland; Molecular Systems Biology Unit, European Molecular Biology Laboratory, 69117 Heidelberg, Germany; Molecular Systems Biology Unit, European Molecular Biology Laboratory, 69117 Heidelberg, Germany; Department of Bioinformatics, Biocenter, University of Würzburg, 97074 Würzburg,Germany

## Abstract

Viruses are the most abundant biological entities on Earth, yet their global diversity remains largely unexplored. Here, we present VIRE, a comprehensive resource comprising over 1.7 million high- and medium-quality viral genomes recovered from >100 000 publicly available metagenomes derived from samples that cover diverse ecosystems, including host-associated, aquatic, terrestrial, and anthropogenic environments. Using a unified and scalable pipeline, we systematically assembled viral genomes and provided detailed information on genome completeness, taxonomic classification, predicted lifestyle, and host assignment based on CRISPR spacer matches. VIRE contains >89 million predicted viral open reading frames, as well as detailed functional annotations derived from multiple databases. Importantly, VIRE is seamlessly integrated with related microbiome resources such as SPIRE (https://spire.embl.de) and Metalog (https://metalog.embl.de), enabling users to jointly explore viral genomes, metagenome-assembled genomes, and associated environmental or clinical metadata. Accessible at https://vire.embl.de, VIRE provides an open-access, scalable platform for investigating viral diversity, evolution, and ecology on a planetary scale.

## Graphical Abstract

**Graphical Abstract:**
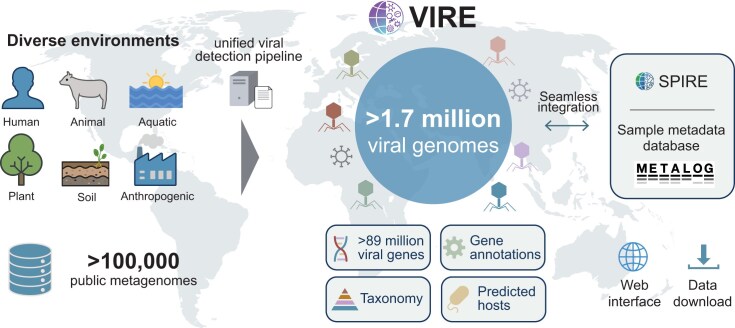

## Introduction

Viruses are estimated to number around 10^31^ particles on Earth, making them the most abundant biological entities on the planet [1, 2]. Among them, bacteriophages, viruses that infect bacteria, are now recognized as key players in microbial ecosystems. Phages shape microbial community structures [3, 4], facilitate horizontal gene transfer between bacteria [5, 6], and drive biogeochemical cycles on Earth [7–9]. Despite their ubiquity and ecological importance, our understanding of viral diversity has remained limited, largely due to the constraints of cultivation-based techniques. The advent of high-throughput sequencing technologies, particularly shotgun metagenomics, has revolutionized our ability to explore viral diversity directly from environmental samples [10–12]. Over the past decade, metagenomic analyses and improved bioinformatic pipelines have uncovered an enormous diversity of previously unknown viral genomes from a wide range of environments, including the human gut [13–18], the ocean [19–24], and soil [25–28]. Yet these newly discovered genomes likely represent only the tip of the iceberg among a vast, largely unexplored viral “dark matter” across Earth’s ecosystems. Understanding the genetic and ecological diversity of such environmental viruses is crucial for understanding viral function, evolution, and host-virus dynamics [29–31]. Moreover, characterizing viral reservoirs in natural environments contributes to pandemic preparedness by providing baseline data for identifying emerging zoonotic threats [32, 33]. To catalog the diversity of uncultivated viruses, previous studies have developed specialized viral genome databases from metagenomic datasets. However, most existing resources are environment-specific (e.g. human gut [13–15, 34], marine [21, 23, 35], or soil [25, 26, 36]) with only a few exceptions [37, 38].

Here, we present VIRE (Viral Integrated Resource across Ecosystems), a global-scale resource of viral genomes assembled from over 100 000 publicly available metagenomic samples spanning diverse environments. VIRE contains >1.7 million medium- to high-quality viral genomes reconstructed through a unified bioinformatics pipeline, making it the largest viral genome database to date. Each genome is accompanied by a rich set of metadata, including taxonomic classification, predicted host organisms, predicted lifestyle (lytic or temperate), and gene annotations derived from multiple functional databases. Importantly, VIRE is seamlessly linked with complementary resources such as SPIRE (https://spire.embl.de) [39] and Metalog (https://metalog.embl.de) [40], allowing users to access associated metagenome-assembled genomes (MAGs) and manually curated metadata of metagenomes, respectively. VIRE provides a comprehensive and scalable platform for exploring global viral diversity, serving as a valuable resource for virology, microbiome research, and microbial ecology.

## Materials and methods

### Identification of viral sequences from metagenomes

The core dataset of VIRE was constructed from a total of 101 623 metagenomic samples derived from 732 independent studies. The majority of these datasets were originally used in the SPIRE resource [39] and consist of publicly available shotgun metagenomes downloaded primarily from the European Nucleotide Archive (ENA) [41] or the Sequence Read Archive (SRA) [42], covering a wide range of environmental samples. The datasets were collected through a semi-automated process and manually curated to exclude certain data types, such as those from artificial experimental systems (e.g. *in vitro* mock communities, laboratory mice, or pathogen challenge studies), as well as amplicon-based and isolate-derived sequences. Moreover, additional virome samples specifically enriched for virus-like particles (VLPs, excluded from SPIRE) were incorporated into the dataset. Each metagenomic sample was annotated with a standardized environmental ontology called microntology, which assigns at least one of 92 terms describing the habitat of the associated microbial community [39].

Metagenomic reads were assembled using MEGAHIT v1.2.9 [43], generating contigs (*n* = 24 883 275 724). For all samples except newly added ones, we used assemblies that had already been generated during the construction of the SPIRE database [39], while newly added virome samples were assembled *de novo* in this study. Contigs longer than 5 kb from bulk metagenomes and those longer than 2 kb from virome metagenomes (*n* = 346 532 161) were subjected to viral detection using geNomad v1.5.2 [44] and CheckV v1.0.1 (database version 1.4) [45]. Contigs with a viral score of ≥0.7 by geNomad and classified as at least medium-quality by CheckV, defined as completeness ≥50% and contamination <10% [46], were considered putative viral genomes (*n* = 1 778 826). Viral sequences with a CheckV kmer_freq score ≥2 (indicative of possible concatemeric repeats) were excluded (*n* = 635). To further improve specificity, Barrnap (https://github.com/tseemann/barrnap) was used to screen for bacterial ribosomal RNA genes (5S, 16S, and 23S ribosomal RNAs), which are rarely found in viral genomes, and contigs encoding any of these genes were removed (*n* = 5049). All data processing steps were implemented in a Nextflow pipeline [47], ensuring reproducibility and scalability.

### Collection of viral genomes from GenBank and RefSeq

To obtain a set of high-confidence viral genomes with reliable taxonomic classification, we downloaded viral genomes labeled as “complete genome” and taxonomically annotated in the International Committee on Taxonomy of Viruses (ICTV) [48] Release 40 from GenBank (accessed in July 2025; *n* = 12 395) [49]. For segmented viruses, such as influenza viruses, individual genome segments were concatenated into a single sequence using a string of ten “N” nucleotides as separators. In addition, we retrieved viral genomes that were not included in the above but were registered as viral genomes in RefSeq (accessed in July 2025; *n* = 8052) [50]. These genomes were processed in the same manner as metagenome-derived viral genomes as described below.

### Clustering viral sequences into species- and genus-level groups

All viral genomes were clustered into species- and genus-level groups using vclust v1.2.2-b687638 [51]. Clustering was performed at 95% and 70% average nucleotide identity (ANI) and 85% alignment fraction (AF) with the Leiden algorithm for species-level and genus-level clusters, respectively, following current guidelines proposed by the ICTV [48].

Rarefaction curves for each environment were generated by progressively subsampling increasing proportions of the full viral genome dataset (10%, 20%, …, 100%). For each sampling fraction, genomes were randomly sampled without replacement 10 times, and the resulting numbers of species-level (>95% ANI) and genus-level (>70% ANI) clusters were calculated. The mean values across the 10 iterations were then plotted to produce the curves.

The species discovery coefficient (α) was calculated for each environment following the approach described previously [52]. In brief, we first determined the number of newly discovered species from the rarefaction analysis for successive increments of sampling effort. We then fitted a log–log linear regression model relating the number of newly discovered species to the cumulative number of species observed, and calculated α as the regression slope plus one.

To evaluate the novelty of genomes in VIRE, we compared viral genomes in VIRE with those from IMG/VR v4 (2022-12-19_7.1) [37]. Because IMG/VR contains low-quality genomes, only those annotated as medium-quality, high-quality, or complete (*n* = 1 059 662) were included for comparison. Clustering was performed using vclust with the Leiden algorithm under the thresholds of ANI > 95% and AF > 85%.

### Host, gene, and lifestyle annotations

To infer bacteriophage host, we employed a CRISPR spacer–based method designed to minimize false positives [53]. We extracted CRISPR spacers from ~1.2 million MAGs from the SPIRE resource (*n* = 9 510 889) [39] and ~1.0 million isolate genomes from the proGenomes v3 database (*n* = 18 937 140) [54] using minced (https://github.com/ctSkennerton/minced). To reduce misbinning-derived contamination, additional filtering was applied to spacers derived from MAGs: for each contig containing a CRISPR locus, genes were predicted and aligned using DIAMOND v0.9.19.120 [55] against the reference gene set of the representative species in SPIRE. If fewer than 50% of genes matched any other MAG of the same genus (excluding self), the spacer was discarded. Spacers derived from contigs shorter than 10 kb were also excluded. The resulting filtered spacers from SPIRE (*n* = 5 702 293) and proGenomes (*n* = 18 937 140) were then aligned to the viral genome sequences using BLASTN v2.5.0 [56], allowing only perfect matches or alignments with a single mismatch or indel under a >95% AF. When a CRISPR spacer matched a viral genome under these criteria, the host taxonomy was assigned according to the GTDB-Tk classification v2.4.0 [57] based on release 220 of GTDB [58].

Protein-coding genes were predicted from the identified viral genomes using prodigal-gv v2.11.0 [44, 59], an algorithm optimized for viral gene calling. Functional annotations were then assigned using eggNOG-mapper v2.1.13 [60], MetaCerberus v1.4.0 [61], and RGI v5.2.1 [62]. These tools provided annotation across multiple databases and functional categories, including eggNOG orthology [63], KEGG Orthology [64], COG [65], PHROG [66], pVOG [67], Pfam [68], TIGRFAM [69], dbCAN [70], and antibiotic resistance genes [62]. To identify auxiliary metabolic genes (AMGs), we used the previously curated set of KEGG orthology terms [71] and calculated the proportion of genes assigned to AMGs relative to the total number of genes in each environment. These proportions were then summarized by functional category for metabolism according to the KEGG database.

The lifestyle (lytic or temperate) of each phage genome was predicted using BACPHLIP v0.9.3 [72], and those with a score of >0.8 were treated as temperate phages. Information on the genetic code of each viral genome was obtained from geNomad and used in downstream analyses.

## Results

### Overview of the viral genomes in VIRE

**Figure 1.**
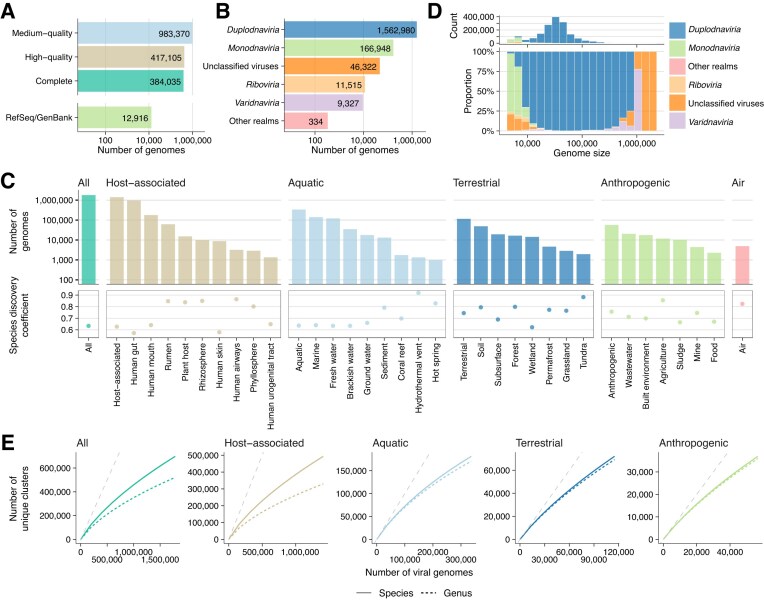
Number of viral genomes included in VIRE. (**A**) Bar plot showing the number of metagenome-derived viral genomes by quality category, as assessed by CheckV, together with viral genomes from RefSeq/GenBank. (**B**) Bar plot showing the number of viral genomes by predicted viral realm, based on classification by geNomad. (**C**) Bar plot showing the number of viral genomes by environment. Each metagenomic sample was annotated with microntology terms, and the number of viral genomes was aggregated based on the presence of each keyword. (**D**) Histogram (top) and stacked bar plot (bottom) showing the distribution of viral genome lengths, with colors indicating the predicted viral realms. For clarity, viral genomes shorter than 5 kb were excluded from the plot. (**E**) Rarefaction curves of viral genomes. All viral genomes were clustered at 95% ANI and 85% ANI to define species- and genus-level groups, respectively. For each environment, genomes were randomly subsampled 10 times, and the average number of recovered species/genus-level clusters was plotted. Dashed gray lines indicate the 1:1 line (diagonal) for reference.

The VIRE database primarily consists of 1 784 510 medium- or high-quality viral genomes (defined as at least >50% completeness and <10% contamination) reconstructed from a total of 101 623 publicly available bulk and virome (VLP-enriched) metagenomic datasets (Supplementary Fig. S1). Quality assessments by CheckV [22] classified these as 384 035 complete, 417 105 high-quality, and 983 370 medium-quality genomes (Fig. 1A). In addition to these metagenome-derived sequences, VIRE includes 12 916 viral genomes downloaded from RefSeq/GenBank [49, 50] (Fig. 1A). Taxonomic classification with geNomad [44] revealed that the majority of sequences (87.2%) belong to *Duplodnaviria*, a realm that encompasses tailed double-stranded DNA bacteriophages (Fig. 1B). This is followed by *Monodnaviria* (9.2%), comprising single-stranded DNA (ssDNA) viruses; unclassified viruses (2.6%); and *Varidnaviria* (0.5%), which includes giant viruses. At the order level, *Petitvirales, Tubulavirales*, and *Sanitavirales* (*Monodnaviria*) and *Crassvirales* and *Autographivirales* (*Duplodnaviria*) were the most abundant (Supplementary Fig. S2). Environmental annotations using the microntology [39] indicated that the majority of metagenome-derived viral genomes (n = 1410837, 78.5%) originate from host-associated environments, most of which were human gut samples (n = 950399, 52.9%) (Fig. 1D). These are followed by viruses derived from aquatic (n = 336505, 18.7%), terrestrial (n = 115044, 6.4%), and anthropogenic environments (n = 56644, 3.2%). The average genome size of these viruses was 38.2 kb (Fig. 1C), and the largest metagenome-derived genome identified was an 836.2 kb phage genome from a bovine sample, classified within *Duplodnaviria*. This genome is among the largest phage genomes reported to date, comparable in size to previously described megaphages (e.g. 841 and 852 kb genomes) [73, 74].

All viral genomes in VIRE were clustered into species- and genus-level groups, operationally defined as genome clusters sharing 95% and 70% ANI, following current ICTV recommendations [48]. This resulted in 706 281 non-redundant species-level and 527 020 genus-level representative sequences. The largest species-level cluster corresponded to phiX174, a bacteriophage genome commonly used as a spike-in for Illumina sequencing quality control. This cluster was detected across diverse environments, including host-associated, aquatic, terrestrial, and anthropogenic samples, suggesting that this control DNA sequence is often incompletely removed from metagenomic datasets before deposition into public archives [75].

Rarefaction analysis revealed that the number of species- and genus-level clusters continued to increase with additional viral genomes across all environments (Fig. 1E and Supplementary Fig. S3), consistent with previous studies [37]. To further quantify this, we calculated species discovery coefficients from the exponent of power laws fitted to rarefaction curves, as described previously [52]. Species discovery coefficients typically range from 0 to 1, where values closer to 1 indicate that rarefaction curves are far from saturation and additional sampling will continue to reveal new species, while values near 0 indicate that diversity has already been largely captured and the discovery of novel lineages is slowing down. In our analyses, high coefficient values (>0.6) were observed for most environments (Fig. 1D). In particular, hydrothermal vent, tundra, and human airways samples all showed coefficients of ≥0.8, indicating that additional sampling effort is expected to discover new lineages at nearly unmitigated rates in these habitats. Other environments, such as agriculture, rhizosphere, rumen, plant host, hot spring, and air, also displayed high coefficients. The lowest coefficient was observed for the human gut and skin, likely reflecting the relatively high sampling effort and relatively low alpha diversity of their microbial community [76], respectively. Nevertheless, even in these environments, the coefficients remained above 0.5, suggesting that the rarefaction curves are still far from saturation. These results indicate that viral diversity across many environments remains substantially undersampled and highlight the need for continued expansion of metagenomic sampling efforts.

When we clustered the viral genomes from VIRE and those from IMG/VR v4 [37], the largest environmental viral genome database to date, at 95% ANI, we identified a total of 1 011 171 species-level clusters (Supplementary Fig. S4A). Of these, 56.1% were unique to VIRE. Compared with IMG/VR alone, VIRE effectively doubled the number of known species-level clusters. Furthermore, when comparing the proportion of viral genomes unique to VIRE across different environments, samples from rumen, coral reefs, built environments, and wastewater showed over 80% unique genomes not represented in IMG/VR (Supplementary Fig. S4B and C). In contrast, the human gut had the lowest proportion of unique genomes among host-associated environments. However, even in this well-studied environment, ~42% of genomes were not present in IMG/VR. Other than the human gut environment, wetland, subsurface, and groundwater had lower proportions of unique genomes (Supplementary Fig. S4B and C). These findings demonstrate that VIRE contains novel viral genomes from a wide range of environments, including the extensively studied human gut.

### Host annotation of phage genomes

Host annotation for phages in VIRE was performed systematically using CRISPR spacer–based predictions, a method recognized for its high specificity and low false-positive rate [53]. CRISPR spacers were extracted from ~1.2 million bacterial/archaeal MAGs derived from the same set of metagenomic samples used for viral genome detection in VIRE, as included in the SPIRE resource [39], and an additional 1.0 million reference genomes from the proGenomes database [54], constituting the largest CRISPR spacer collection to date. These spacers were aligned to viral genomes using stringent matching criteria, resulting in host assignments for 46.8% of all viral genomes in the VIRE database. The predicted host organisms spanned 52 phyla, including both bacteria and archaea, and encompassed a total of 2367 genera, as defined by the GTDB taxonomy [58]. Among viruses with at least one predicted host, ~40.7% were assigned to two or more host genera, potentially representing broad-host-range phages as reported in recent studies [77, 78]. When stratified by environment, host-associated samples yielded the highest proportion of host-annotated viruses (57.4%), followed by those from anthropogenic (13.7%), terrestrial (8.5%), and aquatic samples (3.8%). The assigned host taxonomy was largely consistent with the bacterial taxonomies in the environment. For example, among host-associated viruses, the most frequently predicted hosts were *Faecalibacterium* spp., *Bacteroides* spp., and *Phocaeicola* spp., all of which are common and abundant members of the human gut microbiome. Moreover, at the phylum level, there was a strong positive correlation between the number of genomes/CRISPR spacers included in SPIRE/proGenomes and the number of viral genomes assigned to each phylum (Pearson’s *r* = 0.83 and 0.86, respectively, Supplementary Fig. S5).

**Figure 2.**
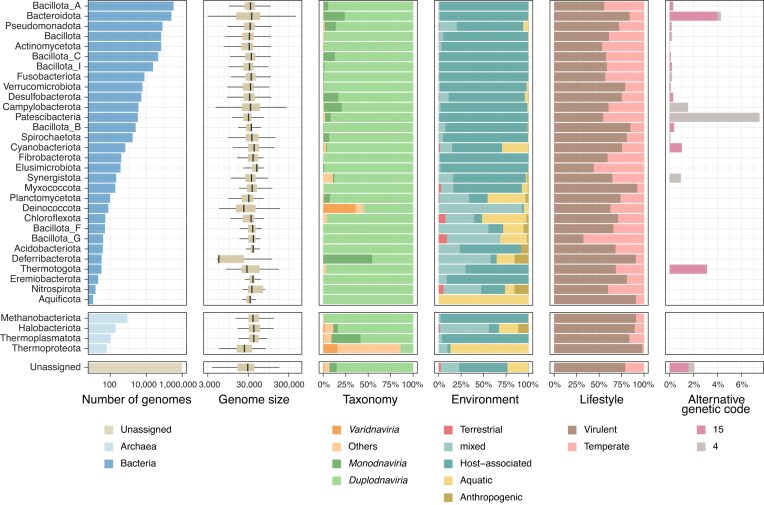
Prokaryotic host annotations for viruses in VIRE. Summary of viral features by predicted bacterial or archaeal host phylum. From left to right, the panels show: the number of viral genomes predicted to infect each phylum, genome size distribution, predicted viral taxonomy from geNomad, environmental source of the metagenomic samples, predicted viral lifestyle, and proportion of viruses predicted to use non-standard genetic codes, assessed by geNomad. Prokaryotic hosts were predicted by mapping CRISPR spacers derived from SPIRE MAGs and proGenomes reference genomes to the viral genomes. Taxonomic assignments for the MAGs and reference genomes were based on GTDB-Tk.

When viral genomes were classified according to the predicted bacterial or archaeal host phyla, several distinctive patterns were observed (Fig. 2). Among viruses predicted to infect members of the Bacteroidota phylum, 4.0% were inferred to use genetic code 15 instead of the standard bacterial genetic code 11. Most of these viruses belonged to the *Crassvirales* order, a dominant viral group in the human gut that infects *Prevotella, Bacteroides*, and *Phocaeicola* (Supplementary Fig. S6). This observation is consistent with previous reports showing that some phages infecting these gut species have alternative genetic codes [79, 80]. Similarly, 7.5% of viruses predicted to infect the Patescibacteria phylum (formerly known as CPR) were inferred to use genetic code 4. This finding is in line with a prior study suggesting that certain Patescibacteria lineages, such as the Absconditabacterales order, utilize alternative genetic codes [81], indicating possible phage adaptation to host-specific translation systems. While the majority of host-assigned viruses were classified as either tailed bacteriophages (e.g. members of the *Caudoviricetes* order within *Duplodnaviria*) or ssDNA viruses (*Monodnaviria*), an exception was observed for viruses predicted to infect Deinococcota, 35.9% of which were assigned to *Varidnaviria*, a viral realm that also includes eukaryotic viruses. This group included members of the non-tailed *Sphaerolipoviridae* family, which are known to infect *Thermus* species in the Deinococcota phylum and inhabit hot springs [82]. In addition, viruses predicted to infect the Deferribacterota phylum had a small genome size (median size = 5612 bp), due to a relatively high proportion of *Monodnaviria* (54.5%), which are ssDNA viruses with relatively small genomes (∼6 kb). These viruses were predicted to infect *Mucispirillum* spp. inhabiting the guts of rodents and other animals.

### Functional annotation of viral genes

**Figure 3.**
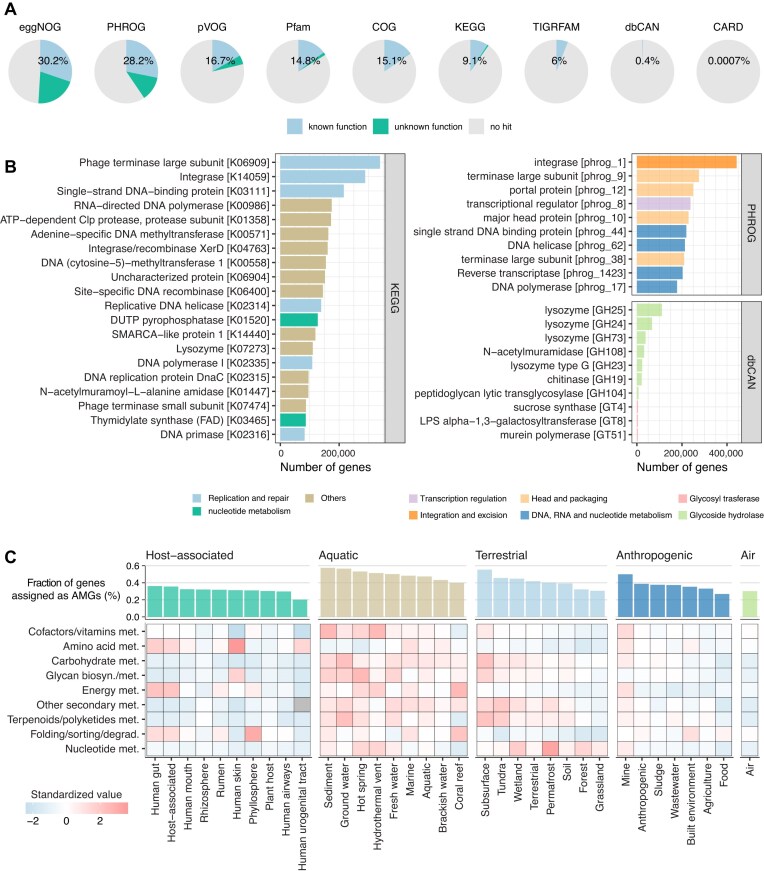
Viral genes and functional annotations. (**A**) Pie chart showing the proportion of viral genes annotated by each functional database. Blue color represents the proportion of genes matching known functions, while green indicates hits to hypothetical or uncharacterized proteins. The numerical values indicate the proportion of genes assigned to known functions. (**B**) Bar plot representing the number of functional annotations derived from KEGG, PHROG, and dbCAN databases. The top 20 functions from KEGG and the top 10 functions from PHROG and dbCAN are displayed. (**C**) Heatmap illustrating the distribution of AMGs across environments. Curated KEGG orthology terms [71]　corresponding to AMGs were detected in viral genomes, and the percentage of AMGs in each category was calculated. Colors represent the standardized value (z-score) of the proportion of each AMG relative to the total number of genes in that environment. The bar plot above shows the percentage of genes assigned as AMGs for each environment.

From ~1.7 million viral genomes, a total of 89 469 781 protein-coding genes were predicted. These genes were comprehensively annotated using multiple functional databases, including eggNOG [63], KEGG [64], COG [65], PHROG [66], pVOG [67], Pfam [68], TIGRFAM [69], dbCAN [70], and CARD [62]. Overall, 40.2% of these genes had at least one hit in any of these databases (Fig. 3A). Among them, eggNOG yielded the largest number of hits, with 51.4% of all viral genes having at least one eggNOG hit, including 27.0% assigned to functionally characterized groups. Consistent with this, eggNOG provided the highest number of unique annotations among the databases (Supplementary Fig. S7). The second-largest number of hits was obtained from PHROG, a database grouping distantly related viral gene families, in which hallmark genes of tailed bacteriophages, such as integrase, terminase large subunit, and portal protein, were frequently identified [66] (Fig. 3B). Additional functional insights were obtained from the broader KEGG annotations, where the most frequently assigned functions included ssDNA-binding proteins, DNA methyltransferases, and DNA polymerases (Fig. 3B). Furthermore, annotations based on the dbCAN database identified lysozymes, viral proteins known to degrade bacterial cell membranes (Fig. 3B). Given that such phage-derived endolysins have potential as alternatives to antibiotics in antimicrobial therapies [83, 84], such annotations may offer valuable information for the rational design of lysozyme-based therapeutics.

Using CARD [62], we identified 618 genes annotated as antibiotic resistance genes, the most abundant being the ACI-1 gene, which confers resistance to cephalosporins. Most viruses carrying this gene had no predicted host, but previous studies have reported that ACI-1 is encoded by prophages in *Negativicutes* inhabiting the human gut [85]. Other detected resistance genes included emrE (from *Escherichia coli*), lnuC, and tet(W/N/W). Of the viruses carrying the resistance genes, 97.2% originated from host-associated samples. Given that host-associated phages account for 78.5% of the VIRE dataset, this represents a statistically significant enrichment of resistance genes in viruses from host-associated environments (Fisher’s exact test, *P* < .01). Nevertheless, the fact that only 618 out of 89 million genes were annotated as resistance genes is consistent with previous studies, which have shown that phages rarely encode antibiotic resistance genes [86].

Phages encode AMGs, which modulate the metabolic functions of their bacterial or archaeal hosts during infection [87, 88]. We examined the distribution of a previously curated set of AMGs [71] in VIRE and found substantial variation in both their abundance and types of AMGs across environments (Fig. 3C). Aquatic viruses, for example, showed a higher proportion of genes assigned as AMGs than those from other environments, spanning diverse functional categories, particularly cofactor and vitamin metabolism, carbohydrate metabolism, and glycan metabolism. Whether the enrichment of AMGs in the aquatic environment simply reflects the greater number of studies conducted in aquatic systems remains to be clarified. In contrast, viruses from host-associated samples, especially those from the human gut, oral cavity, and skin, were enriched in AMGs related to amino acid metabolism and energy metabolism, but had lower frequencies of AMGs associated with carbohydrate and glycan metabolism. Terrestrial viruses from subsurface, tundra, and wetland environments were comparatively enriched in AMGs involved in secondary metabolite ​​biosynthesis and terpenoid/polyketide metabolism relative to the host-associated viruses. These findings suggest that virus–host interactions exhibit environment-specific metabolic signatures, reflecting ecological adaptation between viruses and microbial communities in distinct habitats.

### Seamless integration with other microbiome resources

The metagenomic samples in VIRE use identifiers consistent with those used in our previously developed resources, SPIRE (https://spire.embl.de) [39] and Metalog (https://metalog.embl.de) [40], enabling seamless cross-referencing across the resources. SPIRE is a large-scale microbial genome resource consisting of ~1 million MAGs, allowing users to compare viral genomes and microbial genomes derived from the same metagenomic samples. Metalog is a manually curated metadata repository for metagenomic studies, providing environmental descriptors (including geographic coordinates) extracted from original publications. For human gut samples, Metalog additionally provides host demographic information (e.g. age, sex, geographic origin) and detailed clinical metadata such as disease status and medication use. Additionally, taxonomic profiles of the samples based on mOTUs [89] and MetaPhlAn [90] are also available. By linking viral genomes, microbial MAGs, microbiome taxonomic profiles, and environmental or clinical metadata, VIRE enables large-scale, integrative analyses of microbial ecosystems with unprecedented depth and context.

### Web interface and accessibility

VIRE is publicly accessible at vire.embl.de, where users can browse and download viral genome sequences along with their associated metadata. For each viral genome, the interface provides access to the genome sequence, key quality metrics (e.g. geNomad scores, CheckV completeness, and contamination), predicted genes, functional annotations, host predictions, species- and genus-level cluster assignments, and corresponding metagenome and study metadata. Data can be explored and downloaded by environmental category (host-associated, aquatic, terrestrial, engineered, or human gut) according to the microntology ontology, or by individual study, enabling flexible access tailored to diverse research needs. Community contributions, feature requests, and bug reports are welcome via https://vire.embl.de/contribute.

### Future directions

As the volume of publicly available metagenomic data continues to expand, VIRE will be regularly updated to incorporate newly identified viral genomes. Planned developments include refining viral detection algorithms and gene annotation pipelines to improve the identification of novel viruses and functional elements. Future releases will also integrate long-read metagenomic and metatranscriptomic datasets, broadening the scope to encompass non-tailed phages and RNA viruses. Continued integration with companion microbiome resources such as SPIRE and Metalog will further facilitate comprehensive exploration of viral and microbial ecology across ecosystems and host-associated environments. Over time, the web platform will be enhanced to support more advanced query and analysis capabilities, making VIRE an increasingly powerful tool for the virome research community.

## Discussion

VIRE is a large-scale viral genome resource constructed from over 100 000 publicly available metagenomes using a consistent and standardized pipeline. The underlying metagenomes include a wide range of environments, offering a comprehensive resource for investigating viral diversity on a planetary scale. Each viral genome in VIRE is accompanied by comprehensive annotations, including genome quality metrics, taxonomic classification, predicted host, and gene-level functional annotations, all generated using state-of-the-art tools and databases. These features make VIRE a powerful platform for comparative viromics, host–virus interaction, and the exploration of viral functions across diverse ecosystems. A key strength of VIRE is its seamless integration with other metagenome-based resources, such as SPIRE and Metalog. This interoperability allows users to link viral genomes with MAGs and curated environmental or clinical metadata from the same samples, enabling multi-dimensional analyses of microbial communities in their ecological and host contexts. We anticipate that VIRE will serve as a foundational resource for advancing our understanding of viral diversity, evolution, and ecological roles and will remain a critical resource for the broader microbiome research community.

## Supplementary Material

gkaf1225_Supplemental_File

## Data Availability

All data in the VIRE resource are freely accessible and downloadable via https://vire.embl.de. All metagenomic datasets used for the construction of this database are publicly available through the European Nucleotide Archive (ENA) [41] or the Sequence Read Archive (SRA) [42]. No in-house and unpublished metagenomes were included. VIRE is released under the Creative Commons Attribution-ShareAlike 4.0 International License.
