# Supplementary material for: VIRE: a metagenome-derived, planetary-scale virome resource with environmental context
Source: Nucleic Acids Res. 2025 Nov 29;54(D1):D902–11. doi: 10.1093/nar/gkaf1225 (PMC12807717; doi:10.1093/nar/gkaf1225)
Supplement: gkaf1225_Supplemental_File [file gkaf1225_supplemental_file.pdf]

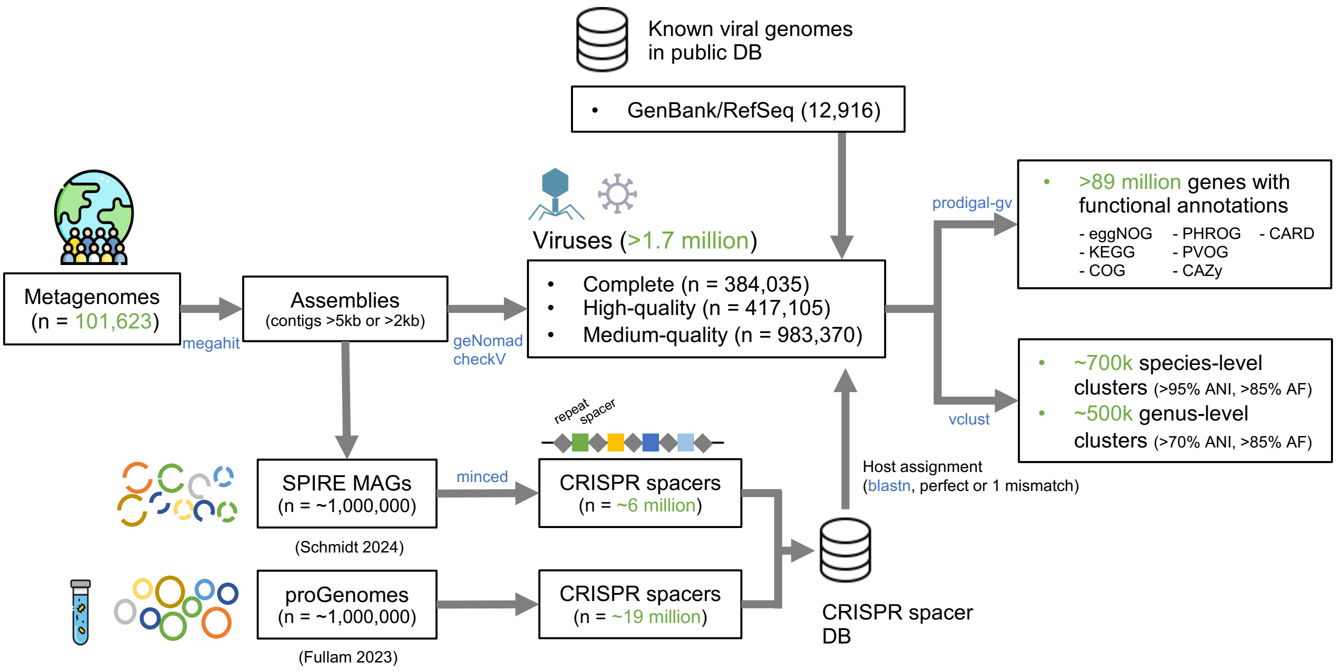

Supplementary Figure 1 | Workflow for viral genome collection in VIRE

A total of 1,784,510 medium- to high-quality viral genomes were detected from 101,623 metagenomic samples. Together with 12,916 viral genomes obtained from GenBank/RefSeq, these constitute the VIRE viral genome dataset (n = 1,797,426). Detailed procedures for each step are described in the Methods section.

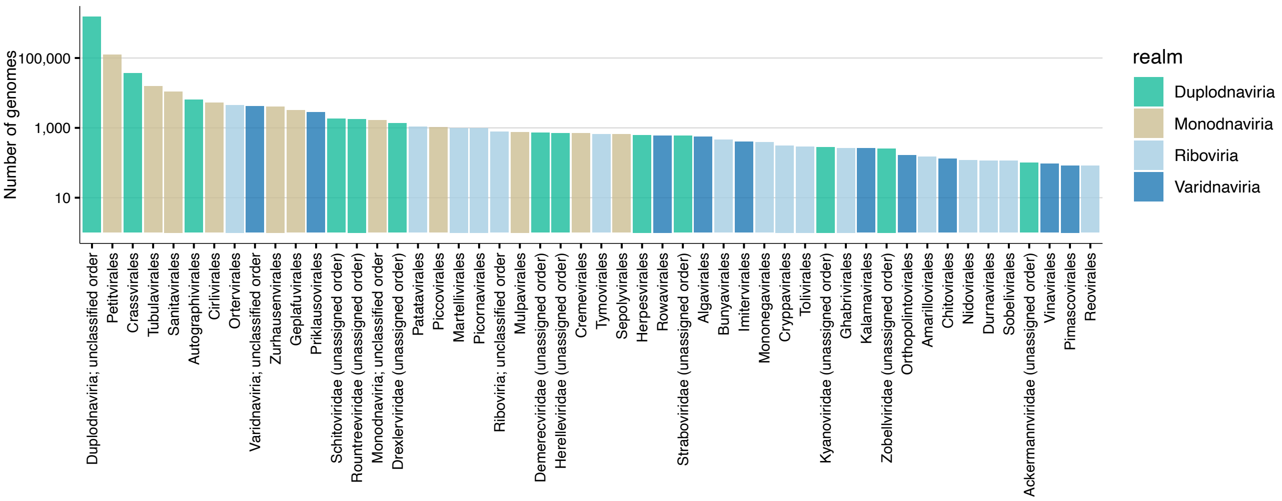

Supplementary Figure 2 | Number of viral genomes at the order level in VIRE

Bar plots show the number of viral genomes classified at the order level by geNomad. For genomes without any assigned order, counts were aggregated as “unclassified order” within each realm. Colors indicate viral realms.

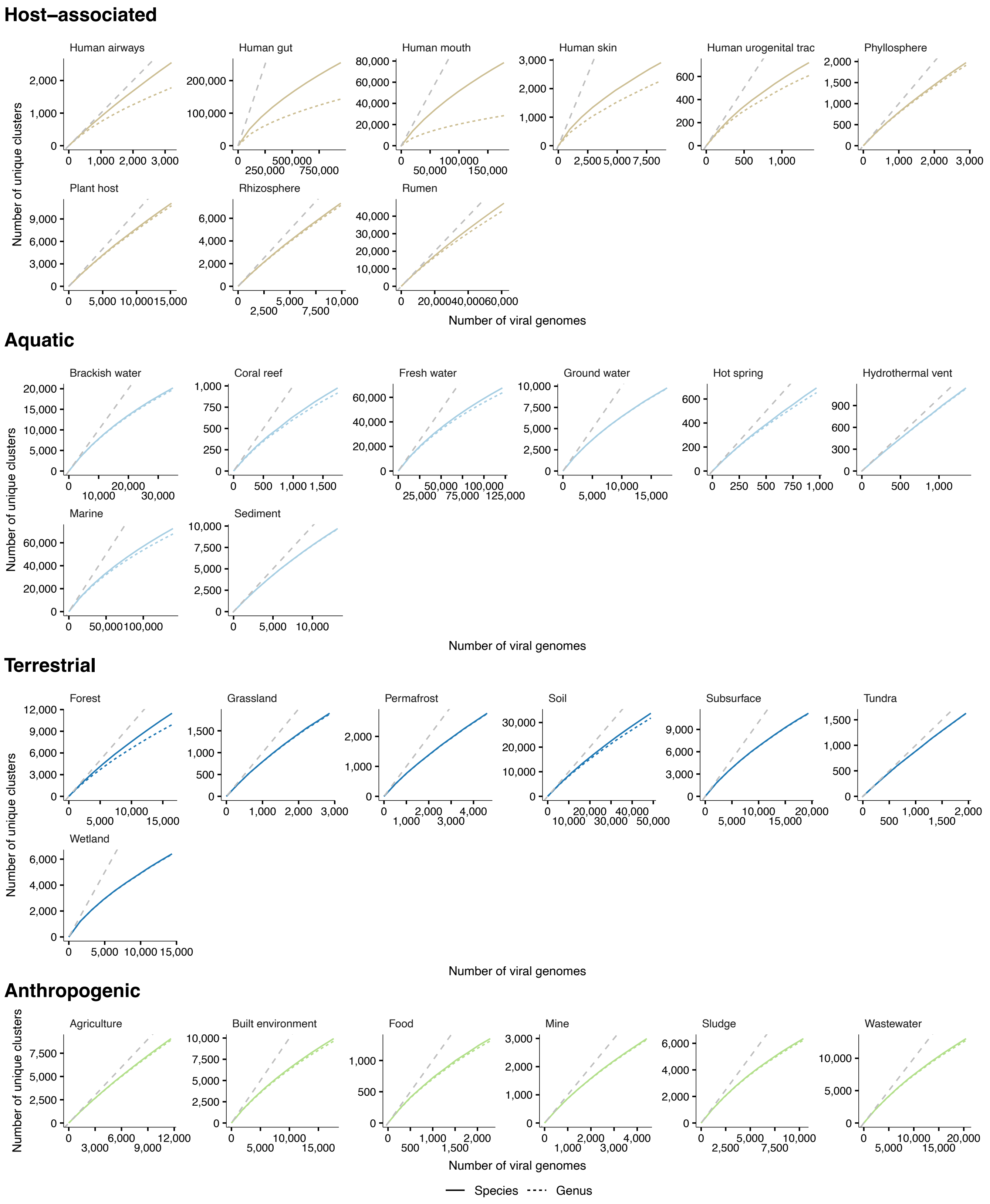

Supplementary Figure 3 | Rarefaction curves of viral genomes for each environment

Solid and dashed lines represent species-level (>95% ANI) and genus-level (>70% ANI) clusters, respectively. The grey dashed line indicates the identity line (y = x). For each environment. The number of genomes was divided into 10 equal-sized bins, and rarefaction curves were estimated by performing 10 random samplings at each bin size.

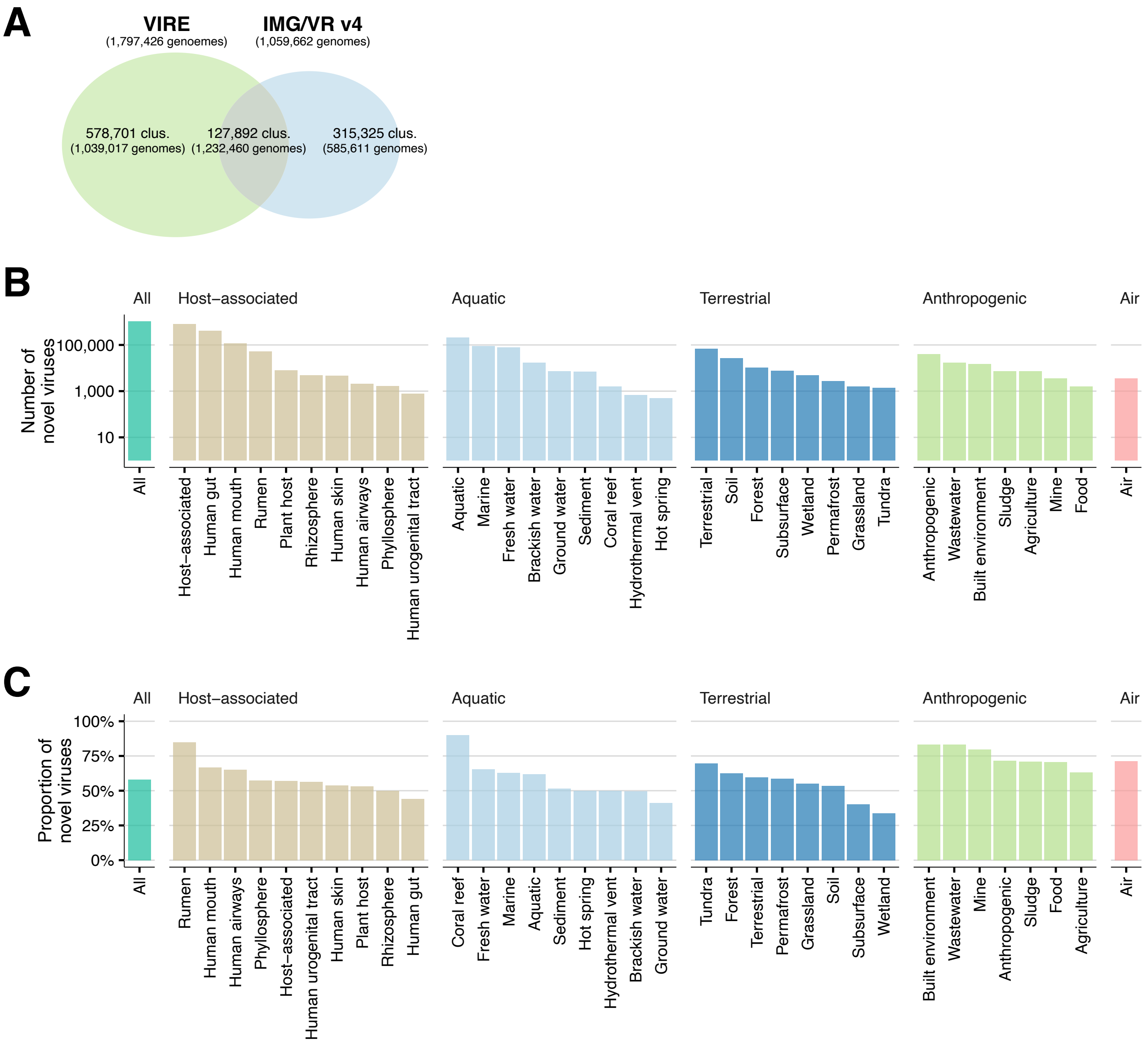

Supplementary Figure 4 | Comparison between VIRE and IMG/VR v4

A, Venn diagram showing clustering results of viral genomes from VIRE and IMG/VR. Medium- or high-quality genomes (n = 1,059,662) from IMG/VR v4 were selected and clustered with VIRE genomes using vclust (>95% ANI and >85% AF). **B, C**, Bar plots showing the number (B) and proportion (C) of genomes in VIRE that did not cluster with any IMG/VR v4 genomes (i.e., novel genomes).

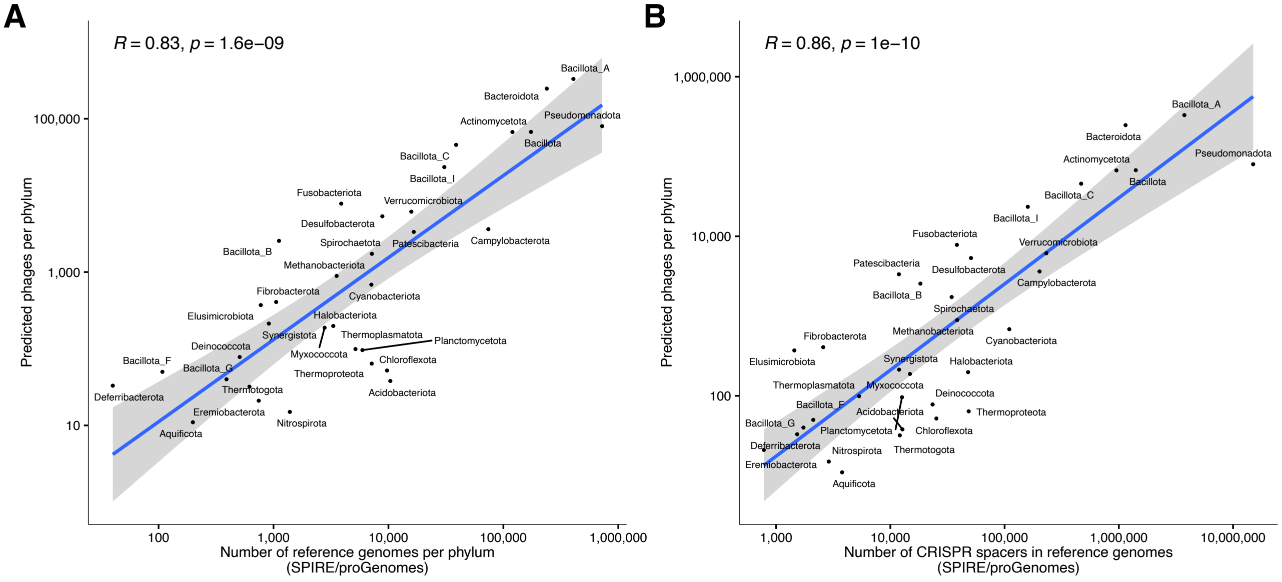

Supplementary Figure 5 | Correlation between the number of reference genomes/CRISPR spacers used for host prediction and the number of phages with predicted hosts

Scatter plot showing the correlation between the number of genomes (**A**) and CRISPR spacers (**B**) per phylum used for host prediction and the number of phages predicted to infect that phylum. The x-axis represents the number of genomes/CRISPR spacers per phylum (from SPIRE/proGenomes) that were used for host prediction, and the y-axis represents the number of phages predicted to infect each phylum. Pearson correlation coefficients were used to evaluate the correlation. 35 phyla that were predicted to be hosts for at least 10 viral genomes are included in the plot.

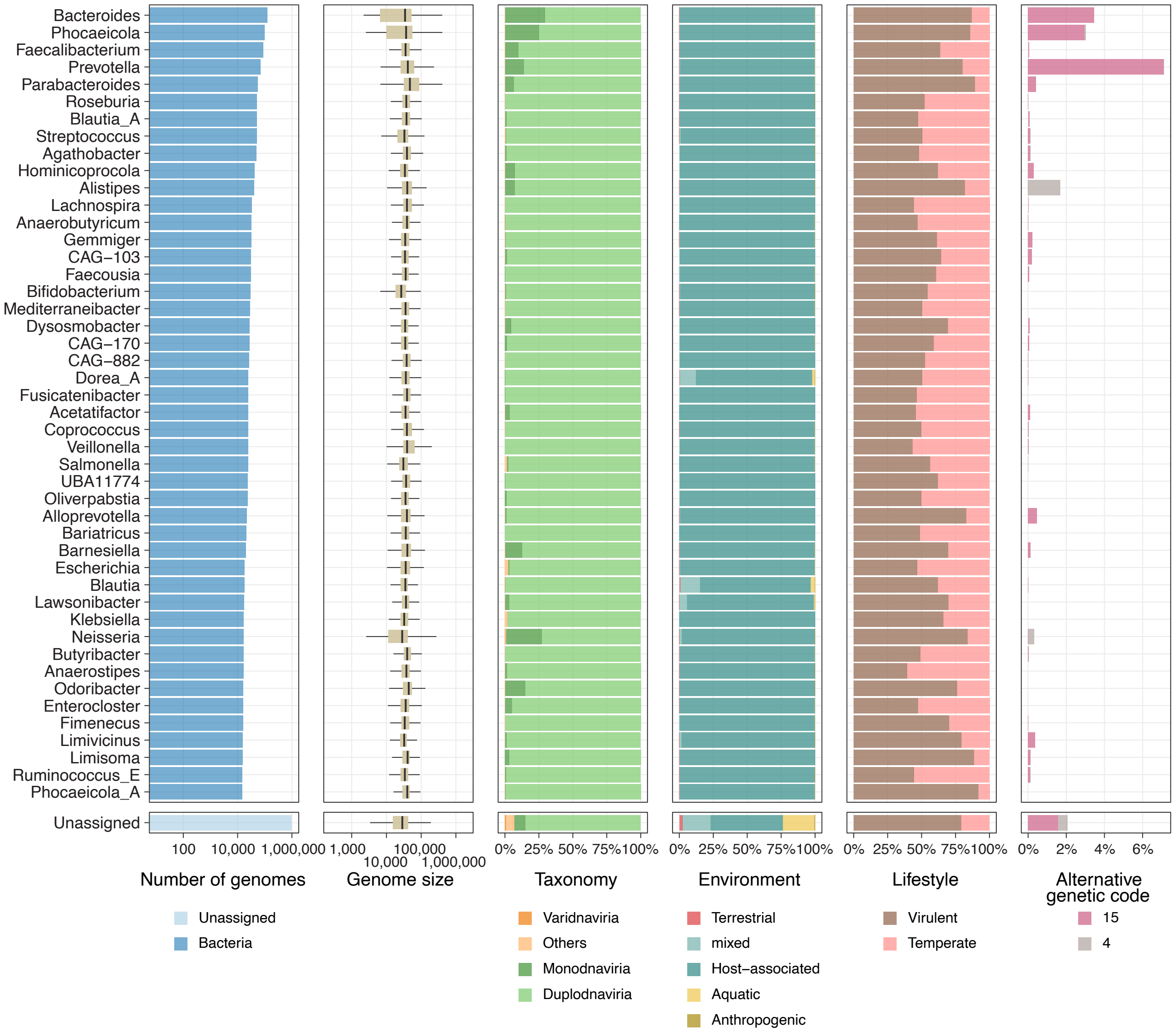

Supplementary Figure 6 | Genus-level prokaryotic host annotations for viruses

Summary of viral features by predicted bacterial or archaeal host genus. From left to right, the panels show: the number of viral genomes predicted to infect each genus, genome size distribution, predicted viral taxonomy from geNomad, environmental source of the metagenomic samples, predicted viral lifestyle, and proportion of viruses predicted to use non-standard genetic codes, assessed by geNomad. Genus-level prokaryotic hosts were predicted by mapping CRISPR spacers derived from SPIRE MAGs and proGenomes reference genomes to the viral genomes. Taxonomic assignments for the MAGs and reference genomes were based on GTDB-Tk.

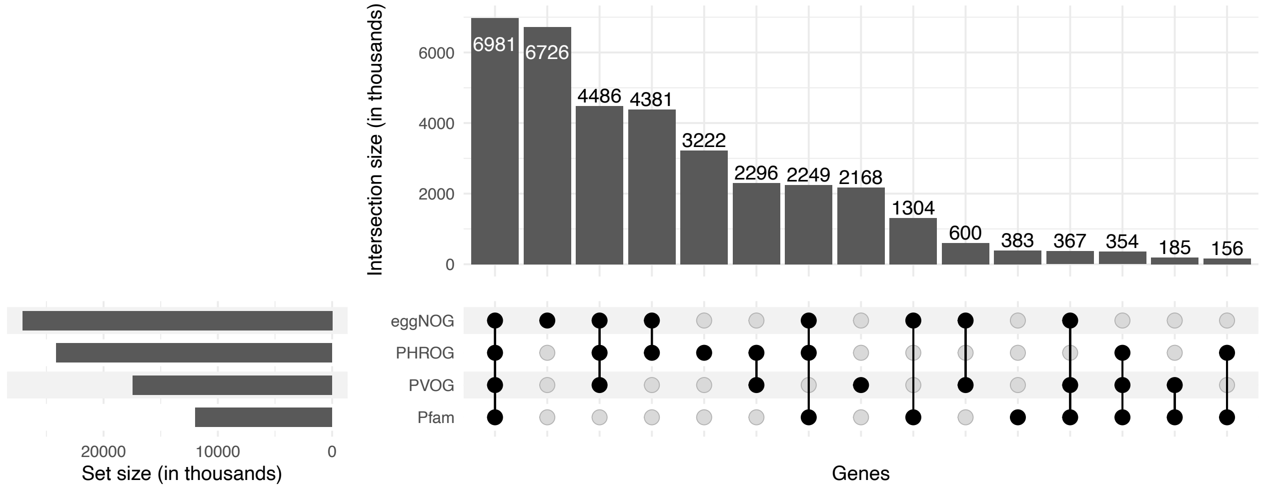

Supplementary Figure 7 | Overlap of viral gene annotations across multiple databases

UpSet plot showing the intersections of viral genes annotated by different reference databases, including Pfam, PVOG, PHROG, and eggNOG. The horizontal bars indicate the total number of genes annotated by each database, while the vertical bars represent the number of genes shared among one or more databases as indicated by the connected dots below. Numbers shown in the plot are given in thousands.
